# Association between Non-Steroidal Anti-Inflammatory Drugs Use and the Risk of Type 2 Diabetes Mellitus: A Nationwide Retrospective Cohort Study

**DOI:** 10.3390/jcm11113186

**Published:** 2022-06-02

**Authors:** Ming-Hsun Lin, Wen-Tung Wu, Yong-Chen Chen, Chieh-Hua Lu, Sheng-Chiang Su, Feng-Chih Kuo, Yu-Ching Chou, Chien-An Sun

**Affiliations:** 1 Division of Endocrinology and Metabolism, Department of Internal Medicine, Tri-Service General Hospital, National Defense Medical Center, Taipei 114, Taiwan; tim6801@msn.com (M.-H.L.); undeca2001@gmail.com (C.-H.L.); doc10504@gmail.com (S.-C.S.); shoummie@hotmail.com (F.-C.K.); 2 Department of Pharmacy, Tri-Service General Hospital, National Defense Medical Center, Taipei 114, Taiwan; allen541312@gmail.com; 3 Data Science Center, College of Medicine, Fu-Jen Catholic University, New Taipei City 242, Taiwan; yongchenchen0824@gmail.com; 4 Department of Medicine, College of Medicine, Fu-Jen Catholic University, New Taipei City 242, Taiwan; 5 School of Public Health, National Defense Medical Center, Taipei 114, Taiwan; trishow@mail.ndmctsgh.edu.tw; 6 Department of Public Health, College of Medicine, Fu-Jen Catholic University, New Taipei City 242, Taiwan

**Keywords:** cohort study, type 2 diabetes mellitus, non-steroidal anti-inflammatory drugs, tramadol

## Abstract

Background: Although the link between non-steroidal anti-inflammatory drugs (NSAIDs) and tramadol and symptomatic hypoglycemia has been documented, there is a limited understanding of the associations of NSAIDs and tramadol with the risk of type 2 diabetes mellitus (T2DM). This study was established to evaluate the association between the clinical use of NSAIDs and the risk of T2DM. Patients and methods: A historical cohort study was conducted using the National Health Insurance Research Database in Taiwan dated from 2000 to 2013. Patients who received NSAIDs for at least 3 prescription orders and without co-treatment of tramadol in the exposure period (from 2000 to 2005) were considered as the exposed cohort (n = 3047). In comparison, patients who received tramadol for at least 3 prescription orders and without concomitant use of NSAIDs in the exposure period were considered as the comparison cohort (n = 9141). The primary outcome was the occurrence of T2DM. Multivariable hazard ratios (HRs) with 95% confidence intervals (CIs) derived from the Cox proportional hazard models were applied to determine the association between NSAIDs use and the risk of T2DM. Results: In the average follow-up period of 9.56 years, there were 159 newly diagnosed T2DM, with an incidence rate of 56.96 per 10,000 person years in the exposed cohort. Comparatively, there were 1737 incident T2DM cases, with an incidence rate of 161.23 per 10,000 person years in the comparison cohort. Compared to the comparison cohort, the NSAIDs cohort showed a significantly reduced risk of T2DM with an adjusted HR of 0.31 (95% CI, 0.26–0.36). Conclusions: Our cohort study provides longitudinal evidence that the use of NSAIDs was associated with a reduced risk of T2DM.

## 1. Introduction

One of the most common complaints for patients to visit doctors is chronic pain. It affects more than 50 million people in the United States [1]. Chronic pain may contribute to psychosocial problems, such as anxiety, depression, and reduced quality of life [2]. Tramadol and non-steroidal anti-inflammatory drugs (NSAIDs) are two major analgesics that widely and commonly used as pharmacologic therapy for chronic pain [3].

NSAIDs are used for analgesics with anti-inflammatory effects through the pathway of COX inhibition that decreased prostaglandin production. NSAIDs are typically divided into selective and non-selective COX-2 groups. The selective COX-2 NSAIDs are commonly prescribed because of less gastrointestinal toxicity compared with non-selective NSAIDs [4]. It has been noted that non-selective NSAIDs may prevent diabetes because of downregulated beta-sheet formation of amylin [5]. Besides, tramadol was a synthetic opioid with blockade of μ-opiate receptors in the CNS with good safety [6]. It was usually used as an alternative analgesic agent to NSAIDs for those with gastrointestinal disorders or renal function impairment. Recent epidemiological studies documented increased risk for symptomatic hypoglycemia among diabetic and nondiabetic outpatients taking therapeutic doses of tramadol [7,8,9]. However, the overall magnitude of the risk of diabetes associated with NSAIDs and tramadol use remains unclear.

The aims of this investigation are to determine whether NSAIDs administration imparts differential risk of type 2 diabetes mellitus (T2DM) as compared with tramadol prescription. Hence, we conducted a population-based historical cohort study to explore the relationships of administrating NSAIDs and tramadol with the risk of new-onset T2DM using data from the Taiwan National Health Insurance Research Database (NHIRD).

## 2. Materials and Methods

### 2.1. Data Sources

The current study was a population-based historical cohort study using medical claims dataset from the National Health Insurance (NHI) Program in Taiwan, the NHIRD. The NHI is a governmental, compulsory-enrollment, single-payer system that had a coverage rate of more than 99% of Taiwanese residents by the end of 2010. The NHIRD contains comprehensive healthcare information, including demographic data of insured individuals, data of clinical visits, diagnostic codes in the format of International Classification of Diseases, Ninth revision, Clinical Modification (ICD-9-CM), and prescription details, as previously described [10]. The NHI administration performs a medical quality monitoring and assurance program every month, including chart reviews, charge audits, and heavy penalties for inappropriate charges or malpractice. Therefore, it is generally believed that these checks and balances can ensure accurate coding and further minimize misclassification error. Thus, the NHIRD can act as a basis for the procurement of high-quality epidemiological studies [11,12], with a good validity on data regarding diagnoses, drug prescriptions, and hospitalizations [13,14]. In the present study, data on medication exposures including NSAIDs and tramadol were historically ascertained from claims data of the NHIRD and then patients were followed up from the initial date of medication prescriptions to the incidence of T2DM. Thus, this is a historical cohort study design. The data of this study were obtained from the Longitudinal Health Insurance Database 2000 (LHID 2000), a subset of NHIRD. The LHID 2000 dataset contains historical ambulatory and inpatient care data from 2000 to 2013 for 1 million randomly sampled beneficiaries enrolled in the NHI system in 2000. There were no significant differences in the distributions of age, sex, and healthcare costs between the individuals in LHID and NHIRD [11,12]. Since the dataset was released for research purposes and the patients included in the dataset had been anonymized, the study was exempt from the need for written informed consent from the subjects. Meanwhile, the present study has been approved by the Institutional Review Board of Fu-Jen Catholic University (FJU-IRB No:C104014).

### 2.2. Study Design and Study Population

Drug use information was obtained from the outpatient pharmacy prescription database. It included prescribed drug dosage, date of prescription, supply days, and total number of pills dispensed. Since patients might discontinue or restart drug therapy, we assumed that patients’ exposure to each studied drug contributed both cumulatively and continuously to themselves. Patients who had ever received NSAIDs or at least 3 prescription orders of tramadol between 1 January 2000 and 31 December 2005 (the exposure period) were identified for the stable NSAIDs users and tramadol users. In this study, we used incident user design [15] to define NSAIDs or tramadol exposures. That is, patients who did undergo NSAIDs or tramadol treatments prior to 2000 were excluded and were naïve users of NSAIDs and tramadol in the study period. Patients who received at least three NSAIDs prescription orders and without any co-prescription of tramadol in the exposure period were considered as the exposed cohort. In comparison, patients who received at least three tramadol prescription orders and without any concomitant use of NSAIDs in the study period were considered as the comparison cohort. Patients who received NSAIDs or tramadol treatments with less than three prescription orders in the study period were excluded. In this study, the temporal period associated with drug exposures was referred to the exposure period, which was equal for each patient. The date of initial use of NSAIDs for each patient was assigned as their index date. Parallelly, the comparison cohort was assigned the same index date as the NHIRD-exposed group. A propensity score was calculated for each patient by using a logistic regression model with covariates of age, sex, index date, baseline comorbidities, including hypertension (ICD-9-CM codes: 401–405), hyperlipidemia (272.4), cardiovascular disease (410–414, 425, 428, 425, 674, and 678), congestive heart failure (428.0), and malignant neoplasms (140–239), as well as use of co-medications, including beta blocking agents [Anatomic Therapeutic Chemical (ATC) code: C07], statins (C10AA01, C10AA02, C10AA03, C10AA04, C10AA05, and C10AA07), and corticosteroids (R01AD) [16,17]. To control for potential confounders between the 2 cohorts, we applied the propensity score as a matching factor at a ratio of 1:3 for exposed and comparison cohorts.

**Figure 1:**
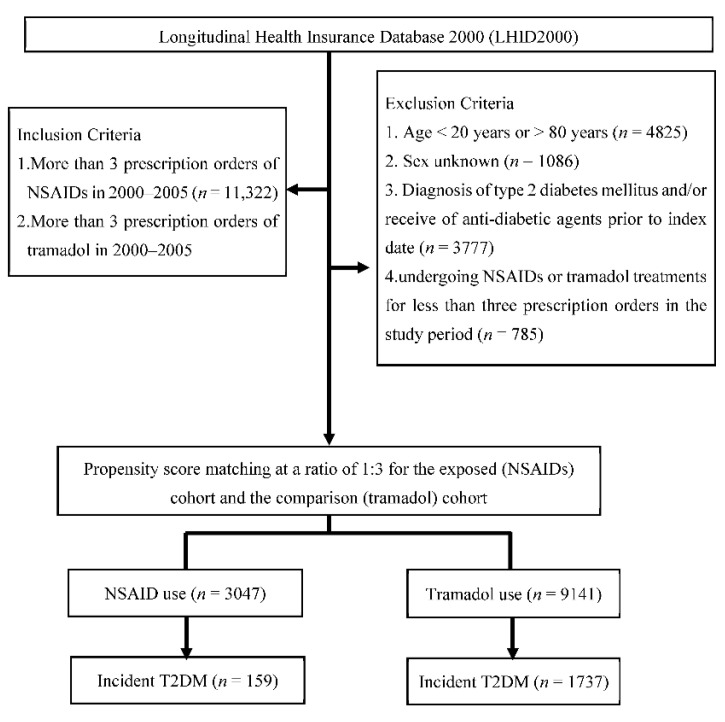
Flow chart of the study design. T2DM, type 2 diabetes mellitus; NSAIDs, non-steroidal anti-inflammatory drugs.

Patients in both exposed and comparison cohorts had no diagnosis of T2DM or prescriptions of anti-diabetic agents prior to the index date. Cohort members were excluded from the study if they were aged <20 years or >80 years, had been diagnosed with T2DM and/or use of anti-diabetic agents prior to the index date, or received NSAIDs or tramadol less than three prescription orders in the study period. We finally included 3047 patients as the exposed cohort and 9141 patients as the comparison cohort (Figure 1).

### 2.3. Clinical Outcomes

The primary outcome was the occurrence of new-onset T2DM. To ensure the diagnostic validity of T2DM, we determined patients having at least 3 outpatient diagnoses of T2DM based on the ICD-9-CM codes of 250.0, 250.1, 250.2, 250.3, 250.4, 250.5, 250.6, 250.7, 250.8, 250.9, accompanied with use of anti-diabetic agents, and excluded the diagnoses of type 1 DM based on ICD-9-CM codes of 250.03, 250.13, 250.23, 250.33, 250.43, 250.53, 250.63, 250.73, 250.83, and 250.93. The date of T2DM diagnosis was based on the date of first diagnosis of T2DM among those consecutive diagnoses.

### 2.4. Covariate Assessment and Adjustment

Patient demographics, baseline comorbidities, and use of co-medications were identified as covariates. We used inpatient and outpatient files to ascertain whether cohort members had aforementioned comorbidities and were determined in a patient if they were diagnosed for any of the aforementioned diseases on at least three outpatient claims or one inpatient claim during the study period. In addition, data on the use of concomitant medications were extracted from the outpatient pharmacy prescription database by using the code of the ATC classification system. These covariates were used to generate propensity scores by logistic regression analyses and the propensity score was included in the regression models for adjustment.

### 2.5. Statistical Analysis

Chi-square and *t*-tests were used to evaluate the differences in distributions of categorical and continuous variables between the study cohorts. In addition, we used the Kaplan-Meier method to estimate the cumulative incidence of T2DM for study cohorts. The log-rank test was used to evaluate differences in cumulative incidence of T2DM between the cohorts. Furthermore, the multivariable Cox proportional hazard regression models were performed to compute hazard ratios (HRs) with 95% confidence intervals (CIs) to assess the associations of administration of NSAIDs and tramadol with the risk of incident T2DM after adjusting for potential confounders. The proportional hazards assumption of the Cox models was evaluated with the log minus log plot of survival and Schoenfeld residuals method, which revealed no significant departures from proportionality in hazards over time (*p* > 0.05) [18]. Defined daily dose (DDD) is the assumed average maintenance dose per day for a drug used for its main indication in adults [19]. To investigate the effect of dose, the cumulative use of NSAIDs was calculated as total prescribed DDD analysis. When a T2DM event occurred, the cumulative NSAIDs dosage was recorded as a total of DDD (cDDD) from drug initiation to the day that the T2DM event occurred. For those who were still at risk (event free and uncensored), the cumulative doses were recorded and ranked at each event time. To examine the dose-response effect of NSAIDs, we categorized the use of NSAIDs into three groups: 0–15 cDDDs, 16–32 cDDDs, and >32 cDDDs. All of the statistical tests were 2-sided, and a level of 0.05 was considered statistically significant. All of the data analyses were performed using SAS software, version 9.1 (SAS Institute, Cary, NC, USA).

## 3. Results

The characteristics of the study cohorts are shown in Table 1. There were no significant differences in the distributions of age, sex, comorbidities, and concomitant medications between the exposed cohort and the comparison cohort due to the propensity score matching schemes.

**Figure 2:**
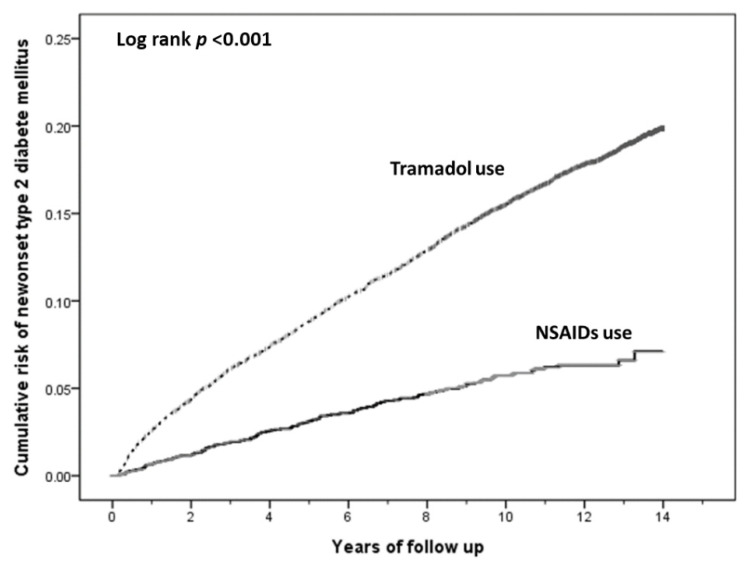
Kaplan-Meier curves for the cumulative risk of incident type 2 diabetes mellitus stratified by the use of non-steroidal anti-inflammatory drugs (NSAIDs) and tramadol with log-rank test.

In the follow-up period of 27,913.40 person years among patients receiving NSAIDs, there were 159 newly diagnosed T2DM, with an incidence rate of 56.96 per 10,000 person years. Comparatively, there were 1737 incident T2DM cases in the follow-up period of 107,731.65 person years among patients undergoing tramadol treatment, with an incidence rate of 161.23 per 10,000 person years. The Kaplan-Meier curves for the cumulative incidence of T2DM among the two cohorts are shown in Figure 2. The cumulative incidence of T2DM was significantly higher in the comparison cohort receiving tramadol than in the cohort who did undergo NSAIDs treatment (*p* < 0.001). Compared to the comparison cohort, the NSAIDs cohort showed a significantly reduced risk of T2DM with an adjusted HR of 0.31 (95% CI, 0.26–0.36). In addition, the risk of T2DM was decreased along with prolongation of drug treatment duration (*p* < 0.001). Compared to patients with drug exposure duration of 1–3215 days, HRs (95% CI) for those with drug exposure duration between 3216–4013 days and ≧ 4014 days were 0.11 (0.07–0.17) and 0.04 (0.01–0.12), respectively. Further, there was a trend toward a reduced risk of T2DM with a higher cumulative dose of NSAIDs prescriptions (*p* = 0.002). Compared to the cDDD of 0–15, HRs (95% CI) for the cDDD of 16–32 and >32 were 0.74 (0.51–1.08) and 0.50 (0.34–0.73), respectively (Table 2).

The stratified analyses based on subgroups formed by sex and age showed results that were consistent with the primary findings, namely, the existence of an inverse association between use of NSAIDs and risk of T2DM after adjusting for potential confounders. Adjusted HRs (95% CIs) were 0.30 (0.25–0.37) and 0.35 (0.27–0.46) for men and women, respectively, as well as 0.33 (0.19–0.55), 0.38 (0.31–0.48), and 0.26 (0.19–0.34) for those aged <40, 40–59, and ≧60 years, respectively (Table 3).

We further evaluated the consistency of the association between administration of NSAIDs and risk of T2DM stratified by baseline comorbidities. Remarkably, the inverse association of the use of NSAIDs and risk of T2DM was consistently observed in each studied comorbidities with HRs (95% CIs) ranging from 0.25 (0.11–0.56) to 0.42 (0.33–0.54) (Table 4).

## 4. Discussion

In the current historical cohort study, our results showed that the administration of NSAIDs was associated with a significantly reduced risk of T2DM. Patients who were undergoing NSAIDs treatment had a 69% lower risk of T2DM than in the comparison cohort receiving tramadol after adjusting for potential confounders. Interestingly, the inverse association between treatment of NSAIDs and the risk of T2DM was consistently present in both sexes and across different age and comorbidities groups.

NSAIDs are one of the most commonly used drug classes in the world [20]. The use of NSAIDs is ubiquitous in rheumatology because of their effectiveness as anti-inflammatory and analgesic agents. In addition to their use in rheumatoid arthritis and osteoarthritis, NSAIDs are widely used in the symptomatic management of other rheumatic diseases characterized by chronic musculoskeletal pain and diverse forms of acute pain. The mechanism of action of NSAIDs involves inhibition of COX-1 and/or COX-2 enzymes. COX-1 catalyzes the production of prostaglandins involved in various physiological functions (for example, the maintenance of renal function, mucosal protection in the gastrointestinal tract, platelet activation) [21]. COX-2 is expressed as part of the inflammatory response, resulting in vasodilation, platelet inhibition, and inhibition of smooth cell proliferation [22]. The inhibition of COX-2 by NSAIDs plays a central role in mediating pain, fever, and inflammation [23]. Animal studies indicated that COX-2 inhibitors can prevent insulin-dependent diabetes by reducing prostaglandin E2 (PGE2) in low-dose streptozotocin-treated mice [24]. In addition, NSAIDs could have an effect on lowering plasma glucose levels by affecting ion channel functions in pancreatic beta cells and, consequently, insulin secretion [25]. More to the point, amylin is secreted from β-cell and regulates the glucose homeostasis with insulin and glucagon secretion. Degeneration of beta-cell due to amyloid deposition with beta-sheet fibrillar amylin was one pathophysiology of T2DM [26]. The study by Thomas et al. found that NSAID and aspirin may prevent amyloid deposition [5]. This is the possible mechanism that NSAID could reduce the risk of diabetes.

Tramadol is a centrally acting analgesic medication that has been in clinical use for several decades. The antinociceptive effects of tramadol are imparted by drug and metabolite binding to μ-opioid receptors and inhibition of neuronal reuptake of serotonin and norepinephrine. Clinically, this may result in decreased blood glucose concentrations [27]. In the present study, patients using NSAIDs had a reduced risk of T2DM compared with those prescribed tramadol. Although previous studies have reported that the use of NSAIDs and tramadol increase the risk of hypoglycemia [7,8,25,28] little is known about the effects of NSAIDs and tramadol administration on the risk of diabetes. Further studies are needed to confirm our findings.

The main strengths of this study include the use of a nationwide comprehensive prescription database rather than self-reported records, thereby minimizing recall bias. In addition, the NHIRD covers a highly representative sample of Taiwan’s general population because the reimbursement policy is universal and operated by a single payer. This allowed for analyses to be performed in a real-life setting in an unselected patient population. However, some limitations to the present study should be mentioned. Indeed, studies that are based on medical claims datasets are often biased because the information on confounders contained in claims datasets is often limited [29]. Thus, several important confounders that are associated with the risk of T2DM, such as smoking habits, physical activity, and obesity, were not available in the NHIRD. Therefore, it cannot be ruled out that there may be residual confounding factors in the present study. Second, the use of prescription database in this study did not permit confirmation of actual usage, as it was impossible to contact patients directly because of the anonymity of the records. The possibility of some degree of treatment noncompliance should also be considered.

## 5. Conclusions

In conclusion, our nationwide population-based cohort study provides longitudinal evidence that the use of NSAIDs was associated with a reduced risk of T2DM. This inverse association between NSAID use and diabetic risk was consistently found across each set of subgroup analyses. Further investigations are needed to determine the clinical implications of the present study.

## Figures and Tables

**Table 1:** Baseline characteristics of study cohorts.

Variable	Study Cohorts		*p* Value
	Tramadol (*n* = 9141)	NSAIDs (*n* = 3047)	
Age group (No., %)			
18 ≤ age < 30	1378 (11.3)	993 (10.9)	
30 ≤ age < 40	1847 (15.2)	1394 (15.2)	
40 ≤ age < 50	2628 (21.6)	2011 (22.0)	
50 ≤ age < 60	2202 (18.1)	1715 (18.8)	
60 ≤ age < 70	2074 (17.0)	1596 (17.5)	
70 ≤ age < 80	2059 (16.9)	1432 (15.7)	
Gender (No., %)			
Female	3959 (43.3)	1257 (41.3)	
Male	5182 (56.7)	1790 (58.7)	
Comorbidities (No., %)			
Chronic liver disease	1340 (14.7)	441 (14.5)	0.801
Malignant neoplasms	2205 (24.1)	702 (23.0)	0.224
Hyperlipidemia	646 (7.1)	215 (7.1)	0.984
Hypertension	3048 (33.3)	981 (32.2)	0.243
Coronary artery disease	1148 (12.6)	408 (13.4)	0.234
Concomitant medications (No., %)			
Beta-blockade	2568 (28.1)	851 (27.9)	0.861
Statins	848 (9.3)	276 (9.1)	0.718
Corticosteroids	4061 (44.4)	1422 (46.7)	0.031

NSAIDs, non-steroidal anti-inflammatory drugs.

**Table 2:** Association between administration of non-steroidal anti-inflammatory drugs (NSAIDs) and risk of type 2 diabetes mellitus (T2DM).

Variable	No. of Subjects	No. of T2DM Cases	Crude HR (95% CI)	Adjusted HR (95% CI)
Overall				
Tramadol	9141	1737	1.00	1.00
NSAIDs	3047	159	0.34 (0.29–0.40)	0.31 (0.26–0.36)
Exposure duration (days)				
1–3215	10154	1873	1.00	1.00
3216–4013	1016	20	0.12 (0.07–0.18)	0.11 (0.07–0.17)
≧4014		3	0.06 (0.02–0.14)	0.04 (0.01–0.12)
*p* for trend	1018		<0.001	<0.001
cDDD				
0–15	10,164	1791	1.00	1.00
16–32	1017	55	0.56 (0.43–0.73)	0.74 (0.51–1.08)
≧32	1007	50	0.34 (0.26–0.46)	0.50 (0.34–0.73)
*p* for trend			<0.001	0.002

cDDD, cumulative defined daily dose; HR, hazard ratio; CI, confidence interval. Hazard ratios were adjusted for age, sex, index date, comorbidities, including hypertension, hyperlipidemia, coronary artery disease, chronic liver disease, and malignant neoplasms, as well as use of concomitant medications, including beta blocking agents, statins, and corticosteroids.

**Table 3:** Association between the use of non-steroidal anti-inflammatory drugs (NSAIDs) and risk of type 2 diabetes mellitus (T2DM) stratified by sex and age.

Variable	No. of Subjects	No. of T2DM Cases	Crude HR (95% CI)	Adjusted HR (95% CI)
Gender				
Male				
Tramadol	5182	1087	1.00	1.00
NSAIDs	1790	101	0.33 (0.27–0.40)	0.30 (0.25–0.37)
Female				
Tramadol	3959	650	1.00	1.00
NSAIDs	1257	58	0.36 (0.27–0.47)	0.35 (0.27–0.46)
Age (years)				
<40				
Tramadol	2387	194	1.00	1.00
NSAIDs	838	15	0.34 (0.20–0.57)	0.33 (0.19–0.55)
40–59				
Tramadol	3726	874	1.00	1.00
NSAIDs	1104	92	0.42 (0.34–0.52)	0.38 (0.31–0.48)
≧60				
Tramadol	3028	669	1.00	1.00
NSAIDs	1105	52	0.26 (0.20–0.35)	0.26 (0.19–0.34)

DM, diabetes mellitus; HR, hazard ratio; CI, confidence interval. Hazard ratios were adjusted for age, sex, index date, comorbidities, including hypertension, hyperlipidemia, cardiovascular disease and malignant neoplasms as well as use of concomitant medications, including beta blocking agents, statins, and corticosteroids.

**Table 4:** Subgroup analysis of the association between administration of non-steroidal anti-inflammatory drugs and risk of type 2 diabetes mellitus on the basis of baseline comorbidities.

Stratified Variable	Crude HR (95% CI)	*p*-Value	Adjusted HR (95% CI)	*p*-Value
Baseline comorbidities				
Chronic liver disease				
Without	0.35 (0.29–0.42)	<0.001	0.33 (0.28–0.40)	<0.001
With	0.27 (0.17–0.45)	<0.001	0.25 (0.15–0.41)	<0.001
Malignant neoplasms				
Without	0.33 (0.28–0.39)	<0.001	0.33 (0.28–0.38)	<0.001
With	0.26 (0.11–0.59)	0.001	0.25 (0.11–0.56)	0.001
Hyperlipidemia				
Without	0.34 (0.28–0.40)	<0.001	0.32 (0.27–0.38)	<0.001
With	0.36 (0.21–0.63)	<0.001	0.37 (0.21–0.63)	<0.001
Hypertension				
Without	0.29 (0.24–0.37)	<0.001	0.28 (0.22–0.34)	<0.001
With	0.43 (0.33–0.55)	<0.001	0.42 (0.33–0.54)	<0.001
Coronary artery disease				
Without	0.34 (0.29–0.41)	<0.001	0.32 (0.27–0.38)	<0.001
With	0.33 (0.20–0.54)	<0.001	0.34 (0.20–0.55)	<0.001

HR, hazard ratios, CI, confidence interval. Hazard ratios were adjusted for age, sex, index date, and use of concomitant medications, including beta blocking agents, statins, and corticosteroids, and mutually adjusted for comorbidities shown in the table.

## Data Availability

All data were deposited in an appropriate public repository. The data on the study population that were obtained from the NHIRD are maintained in the NHIRD in Taiwan (http://nhird.nhri.org.tw/, accessed on 21 February 2022).
